# The Formation of Oregon’s Center of Excellence for Behavioral Health & Aging

**DOI:** 10.1093/geroni/igaf122.1979

**Published:** 2025-12-31

**Authors:** Allyson Stodola, Walter Dawson

**Affiliations:** Portland State University, Portland, Oregon, United States; Oregon Health & Science University, Portland, Oregon, United States

## Abstract

As Oregon’s population rapidly increases, there is an increased need for improved access to behavioral health resources. In 2024, the Oregon Health Authority funded a collaboration of Portland State and Oregon Health & Science University to establish Oregon’s Center of Excellence for Behavioral Health and Aging (OCEBHA) to address growing mental health and substance use disorders among older adults by improving access to behavioral health care services and supports. OCEBHA serves as a backbone organization to support partners in aging, behavioral health, and disability services by advancing workforce development, policy innovation, and translational research. The Center draws on models such as the State-University Partnership Learning Network using a multidisciplinary approach to serious mental illness, substance use, health equity, and best practices in care. Guided by a local steering committee—including individuals with lived experience—and a national advisory board, OCEBHA works to bridge policy and practice through training, resource development, and system-wide improvements.

